# Cognitive impact and brain structural changes in long COVID patients: a cross-sectional MRI study two years post infection in a cohort from Argentina

**DOI:** 10.1186/s12883-024-03959-8

**Published:** 2024-11-18

**Authors:** Sol A. Cataldo, Andrea Micciulli, Laura Margulis, Melina Cibeyra, Sabrina Defeo, Silvina G. Horovitz, Analía Martino, Raul Melano, Milagros Mena, Francisco Parisi, Diego Santoro, Florencia Sarmiento, Martin A. Belzunce

**Affiliations:** 1 Centro Universitario de Imágenes Médicas (CEUNIM), Escuela de Ciencia y Tecnología, Universidad Nacional de Gral. San Martín, Campus Miguelete, 25 de Mayo 901, San Martín, Buenos Aires 1650 Argentina; 2 Unidad de Neuropsicología, Servicio de Neurología, Hospital Interzonal General de Agudos Eva Perón, San Martín, Buenos Aires, 1650 Argentina; 3 Facultad de Psicología, Universidad de Buenos Aires, Ciudad Autónoma de Buenos Aires, Argentina; 4 Servicio de Neurología, Hospital Interzonal General de Agudos Eva Perón, San Martín, Buenos Aires, 1650 Argentina; 5 National Institute of Neurological Disorders and Stroke, National Institutes of Health, Bethesda, MD USA; 6 Escuela de Humanidades, Universidad Nacional de Gral. San Martín, Campus Miguelete, 25 de Mayo y Francia, San Martín, Buenos Aires, 1650 Argentina; 7 Instituto de Ciencias Físicas (ICIFI UNSAM-CONICET), Escuela de Ciencia y Tecnología, Universidad Nacional de Gral. San Martín, Campus Miguelete, 25 de Mayo y Francia, San Martín, Buenos Aires, 1650 Argentina; 8 Center for Complex Systems and Brain Sciences (CEMSC3), Instituto de Ciencias Físicas (ICIFI UNSAM-CONICET), Escuela de Ciencia y Tecnología, Universidad Nacional de Gral. San Martín, Campus Miguelete, 25 de Mayo y Francia, San Martín, Buenos Aires, 1650 Argentina; 9 Consejo Nacional de Investigaciones Científicas y Tecnológicas (CONICET), Godoy Cruz 2290, Godoy Cruz, Buenos Aires, 1425 Argentina

**Keywords:** Long COVID, Neuroimaging, Brain atrophy, Structural MRI, Cognitive dysfunction, Quality of life

## Abstract

**Objective:**

Long COVID is a condition characterised by persistent symptoms after a SARS-CoV-2 infection, with neurological manifestations being particularly frequent. Existing research suggests that long COVID patients not only report cognitive symptoms but also exhibit measurable cognitive impairment. Neuroimaging studies have identified structural alterations in brain regions linked to cognitive functions. However, most of these studies have focused on patients within months of their initial infection. This study aims to explore the longer-term cognitive effects and brain structural changes in long COVID patients, approximately two years post-infection, in a cohort from San Martín, Buenos Aires, Argentina.

**Methods:**

We conducted a cross-sectional study involving 137 participants: 109 with long COVID symptoms and 28 healthy controls. The participants underwent an initial clinical assessment, completed a structured questionnaire and standardised scales, underwent a cognitive assessment, and had a brain MRI scan. Structural MRI images were processed via FreeSurfer and FSL to obtain volumetric measures for subcortical and cortical regions, along with regional cortical thickness. Differences between groups for these variables were analysed using ANCOVA, with permutation tests applied to correct for multiple comparisons.

**Results:**

Long COVID patients reported persistent cognitive symptoms such as memory problems and brain fog, with higher levels of fatigue and reduced quality of life compared to controls. Despite subjective cognitive complaints, cognitive tests did not reveal significant differences between groups, except for the TMT-A (*p* = 0.05). MRI analysis revealed decreased volume in the cerebellum (*p* = 0.03), lingual gyrus (*p* = 0.04), and inferior parietal regions (*p* = 0.03), and reduced cortical thickness in several areas, including the left and right postcentral gyri (*p* = 0.02, *p* = 0.03) and precuneus (*p* = 0.01, *p* = 0.02).

**Conclusions:**

This study highlights the enduring impact of long COVID on quality of life and physical activity, with specific brain structural changes identified two years post-infection. Although cognitive tests did not show clear impairment, the observed brain atrophy and significant reduction in quality of life emphasize the need for comprehensive interventions and further longitudinal studies to understand the long-term effects of long COVID on cognition and brain health.

**Supplementary Information:**

The online version contains supplementary material available at 10.1186/s12883-024-03959-8.

## Introduction

Long COVID, characterised by persistent symptoms after a SARS-CoV-2 infection, has become a pressing global health problem. Estimates suggest that between 10% and one-third of those infected continue to experience symptoms after recovering from COVID-19 [1–3]. The World Health Organization (WHO) defines long COVID as a condition marked by symptoms impacting daily life, such as fatigue, shortness of breath, and cognitive dysfunction, which occur after a history of probable or confirmed SARS-CoV-2 infection (WHO, 2021). Reported symptoms are diverse, frequently including fatigue, dyspnoea, muscle pain, memory loss, attention deficits, memory problems, and decreased psychophysical performance [4–7].

Neurological symptoms are among the most frequently reported in long COVID cases, although they are often based on patient-reported descriptions. Emerging evidence suggests that some of the long COVID patients suffer not only from cognitive symptoms, but also cognitive impairment, particularly in executive functions [8–11]. While cognitive symptoms refer to subjective experiences, cognitive impairment can be objectively assessed through cognitive tests. For instance, Douaud et al. found that patients who had a COVID-19 infection showed cognitive impairment, measured by the completion time of the Trail Making Test A and B, on average 141 days post-infection [12]. Another study reported that 26% of the patients exhibited mild cognitive impairment nine months after infection, assessed with the MoCA [11]. Additionally, a systematic review by Crivelli et al. showed that patients recovered from COVID-19 performed lower in general cognition tasks compared to healthy controls up to seven months post-infection [13].

Several hypotheses have been proposed regarding the mechanisms of long COVID in the brain. First, COVID-19 may directly damage neurons and other brain cells, as it is capable of crossing the blood-brain barrier and reach the CNS [14–17]. By infecting neurons, astrocytes, and vascular endothelial cells in the brain, COVID-19 can damage neurons in the hippocampus and other brain structures, and disrupt neural network connections [18–21], potentially explaining the memory and cognition-related symptoms. Second, COVID-19 infections can trigger a cytokine storm and widespread neuroinflammation, damaging neurons, synapses, and cognitive network connections [22, 23]. Other hypothesis include reduced oxygen exchange due to microclots from hypercoagulation and hyperactivate platelets induce by COVID-19 [24, 25], as well as metabolic and mitochondrial dysfunction [26, 27].

Magnetic resonance imaging (MRI) can measure structural changes associated with the neuronal damage present in most of the hypothesis for the mechanisms of long COVID. A recent systematic review showed several structural changes using brain MRI images, including white matter alterations, grey matter volume, and cortical thickness reductions [28]. One of the first neuroimaging studies on long COVID showed structural brain changes (greater reduction in grey matter thickness and global brain size) in 785 participants from the UK Biobank, aged 51 to 81 years, who underwent magnetic resonance imaging (MRI) scans before and, on average, 141 days after a COVID infection [12]. The reduction in grey matter volume has also been observed in other studies, including specific regions such as the hippocampus and the insular, orbitofrontal, and cingulate cortices [29–31]. These areas are crucial for different cognitive functions such as attention, motivation and decision making, which are commonly affected in COVID-19 patients. As with the evidence of cognitive deficit, these structural changes were observed relatively short-term after a COVID infection. While most studies focus on changes in cognition and brain structure within just a few months of the initial infection, we aim to investigate whether these changes persist in the long term as some patients may experience improvement over time.

In this work, we study the neurocognitive impact of long COVID three years after the emergence of COVID-19 infections. Our aim is to quantitatively assess brain atrophy, cognitive performance in a subset of executive functions, and health-related quality of life in patients who have experienced long COVID symptoms for more than two years on average. To achieve this, we recruited a cohort of patients with long COVID symptoms residing in the suburbs of Buenos Aires, Argentina. These participants underwent MRI, cognitive assessments, and completed a structured questionnaire.

## Methods

### Study design

This was a cross-sectional study involving 137 subjects, consisting of 109 with long COVID symptoms (LC group) and 28 healthy controls living in San Martín city, in the suburbs of Buenos Aires, Argentina (The San Martín Cohort). The inclusion criteria for the LC group were as follows: persistent cognitive symptoms for more than 12 weeks post COVID-19 infection and ages between 35 and 65 years. Patients that had mild, moderate, and severe infections were included. The healthy control group consisted of individuals who had never contracted COVID-19 or who had mild infections and fully recovered within a short period. The exclusion criteria for all participants included ineligibility for an MRI scan and any pre-existing neurological or neuropsychiatric disorders. Demographic data for each group are presented in Table 1.

**Table 1:** Demographic and clinical information of the long COVID and control groups

	Control (28)	Long COVID (109)	*p* value
Sex (female), n(%)*	19(70.4%)	79(72.5%)	0.88
Age (years), mean ± SD†	45.2 ± 9.9	48.4 ± 8.0	0.08
BMI, mean ± SD†	26.5 ± 3.9	28.3 ± 6.1	0.21
Education‡, n(%)*			
Basic/Intermediate/Advanced	3(11.1%) / 12(44.4%) / 9(33.3%)	12(11.0%) / 52(47.7%) / 45(41.3%)	0.76
N/A	3 (11.1%)	0(0%)	
**COVID**			
Confirmed diagnosis*	9(32.1%)	109(100%)	**< 0.01**
Severity^§^, n(%) (Uninfected/mild/ moderate/severe)	19(70.4%) / 8(29.6%) / 0(0%) / 0(0%)	0(0%) / 91(83.5%) / 14(12.8%) / 4(3.7%)	
Days since first infection, mean ± SD *	507.1 ± 289.0	908.2 ± 260.5	
Vaccinated, n(%)	28(100%)	109(100%)	1
Unvaccinated before first infection, n/T(%)^¶^	2/5(7.4%)	71/99(71.1%)	
Health history, n(%)*			
High blood pressure	7(25.9%)	33(30.4%)	0.75
Diabetes	1(3.7%)	6(5.5%)	1
Asthma	0(0%)	8(7.3%)	0.31
High cholesterol	8(29.6%)	28(25.7%)	0.89
Heart attack	0(0%)	1(0.9%)	1
Angina pectoris	0(0%)	1(0.9%)	1
Embolism or thrombosis	0(0%)	2(1.8%)	1
Blood pressure medication	7(25.9%)	25(22.9%)	1
Aspirins	0 (0%)	3(2.8%)	0.87
Antiplatelets	1 (3.7%)	1(0.9%)	0.87
Smoking status, n(%)*			
Yes	9(33.3%)	31(28.4%)	0.20
Ex-smoker	3(11.1%)	29(26.6%)	
No	14(51.9%)	49(45.0%)	
Alcohol use frequency, n(%)*			
Yes, every day	0(0.0%)	7(6.4%)	0.06
Yes, only weekends	15(55.6%)	38(34.9%)	
No	0(0.0%)	0(0.0%)	
**Symptoms (persists / not anymore / never had it), n(%)**			
Memory Problems		**91(83.5%)** / 1(0.9%) / 17(15.6%)	
Fatigue		**86(78.9%**) / 18(16.5%) / 5(4.6%)	
Brain Fog		**87(79.8%)** / 2(1.8%) / 20(18.3%)	
Problems with attention		**86(78.9%)** / 3(2.8%) / 20(18.3%)	
Muscle Weakness		**73(67.0%)** / 18(16.5%) / 18(16.5%)	
Muscle Ache		**55(50.5%)** / 30(27.5%) / 24(22.0%)	
Sleeping Problems		**54(49.5%)** / 4(3.7%) / 51(46.8%)	
Dyspnoea		**52(47.7%)** / 26(23.9%) / 31(28.4%)	
Communication Problems		**48(44.0%)** / 5(4.6%) / 56(51.4%)	
Headaches		**44(40.4%)** / 42(38.5%) / 23(21.1%)	
Anosmia		**7(15.6%**) / 44(40.4%) / 48(44.0%)	

*Chi-square test. †Kruskal Wallis. ‡Education levels defined as the aggregate levels of education presented in ILOSTAT [36]. ^§^ Using the WHO clinical progression scale: uninfected, ambulatory mild disease, hospitalised: moderate disease, hospitalised: severe disease. ^¶^ T is the number of participants with this information available

**Fig. 1:**
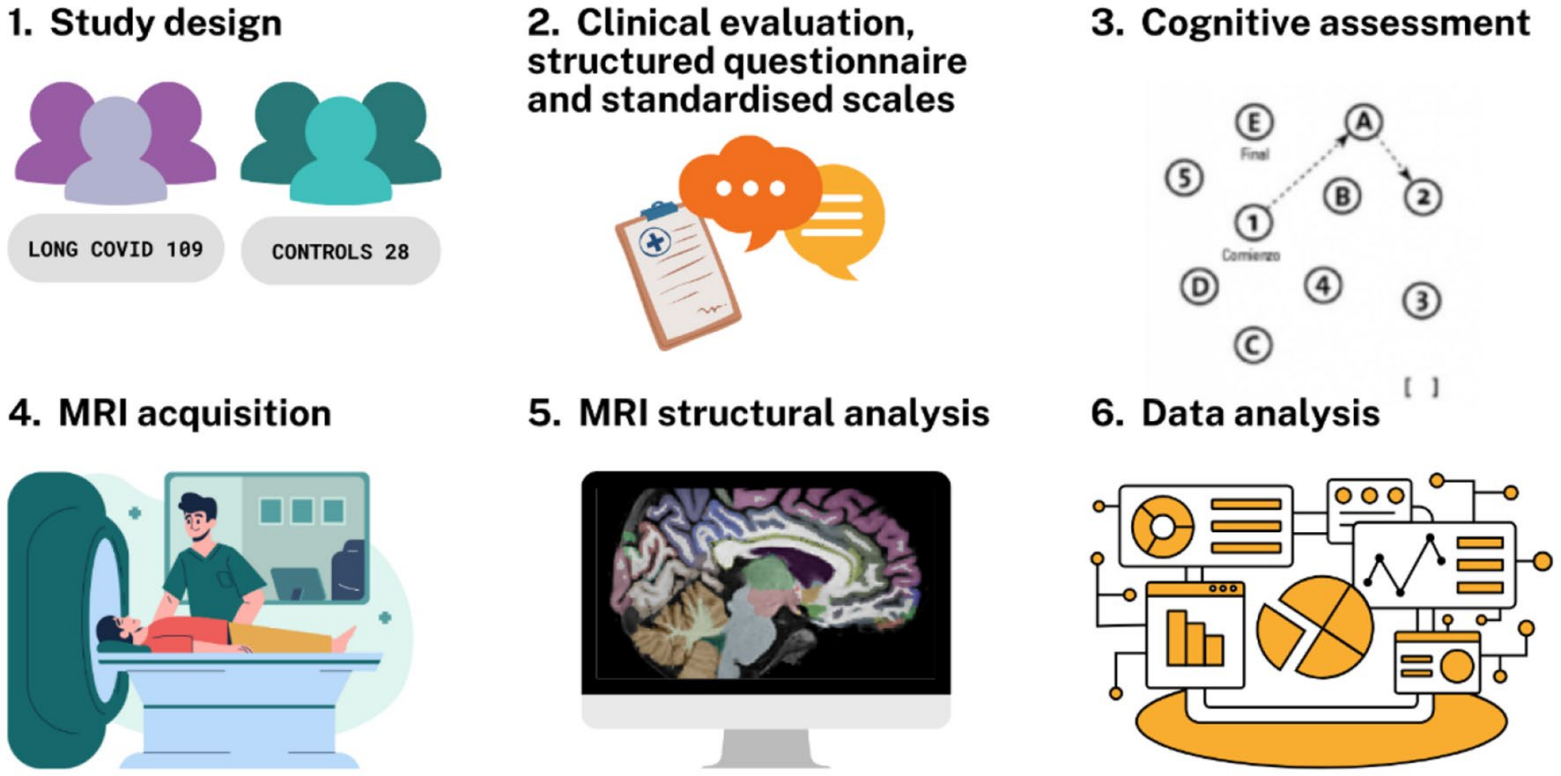
Description of the steps in this study. The San Martín cohort consists of 109 participants with long COVID symptoms and 28 healthy controls (**1**), who underwent an initial clinical assessment with a neurologist, completed a structured questionnaire and standardised scales (**2**), underwent cognitive assessment (**3**), and had a brain MRI scan (**4**). The structural MRI images were processed using FreeSurfer and FSL (**5**) and all the data was statistically analysed comparing both groups

In Fig. 1, we show the steps involved in the acquisition and data analysis of this work. The volunteers underwent an initial clinical assessment with a neurologist, completed a structured questionnaire, underwent a cognitive assessment, and had a brain MRI scan. All subjects provided written informed consent. The study was approved by the Hospital Interzonal General de Agudos (HIGA) Eva Peron Research Ethics Committee (IRB00002792).

### Participant recruitment

Participants were recruited between February and November 2023 through multiple channels to ensure a diverse and representative sample. Recruitment methods included:

- Neurology Clinic at the HIGA Eva Peron Hospital (San Martin City, Buenos Aires, Argentina): Patients visiting the neurology clinic were informed about the study and invited to participate if they met the inclusion criteria.
- Advertisements and social media: The study was promoted through targeted advertisements and social media platforms to reach a broader audience.
- Email Invitations: All patients registered as having tested positive for COVID-19 in San Martín City since the start of the pandemic were contacted by email and invited to participate in the study if they had persistent cognitive symptoms post-infection.

### Structured questionnaire and standardised scales

The structured questionnaire included questions about general health, COVID infection, symptoms, and sociodemographic information. The following symptoms were assessed for persistence: memory problems, fatigue, brain fog, problems with attention, muscle weakness, muscle ache, sleep problems, dyspnoea, communication problems, headaches, anosmia, and loss of taste. A scale to assess the severity of cognitive symptoms was created based on the number of persistent cognitive symptoms each participant reported. The symptoms included in the scale were brain fog, communication problems, memory problems, attention problems, and fatigue. This scale ranges from 0 to 5, where 0 indicates no symptoms and 5 indicates all symptoms.

In addition, the following standardised scales were administered: the Fatigue Assessment Scale (FAS) questionnaire [32] to assess fatigue condition, the Pittsburgh Sleep Quality Index (PSQI) [33] to evaluate sleep quality, the International Physical Activity Questionnaire (IPAQ) [34] assessed physical activity, and the EQ-5D-5L (5-level EuroQol-5 Dimension) [35] described and evaluated health-related quality of life across five dimensions: mobility, self-care, usual activities, pain/discomfort, and anxiety/depression. Severity in each dimension was rated by the participants on a five-point scale from 1 (no problem) to 5 (extreme problem). The EQ-5D-5L also includes a visual analogue scale (EQ VAS) that allowed the participants to rate their overall current health on a vertical scale ranging from 0 (“the worst imaginable condition”) to 100 (“the best imaginable condition”).

### Cognitive assessment

A trained psychologist administered a neuropsychological test battery including: the Trail Making Test Parts A (TMT-A) and B (TMT-B) to evaluate attentional and executive processes [37], the Stroop Color-Word Interference test to measure cognitive flexibility, cognitive inhibition, and information processing speed [38] (which includes word (STROOP W), colour (STROOP C), word-colour (STROOP WC) and interference (STROOP interf) tests), the Digit Span Forward (DGS-F) and Backward (DGS-B) tasks to evaluate verbal attention span and working memory [39], and the Montreal Cognitive Assessment (MoCA) as a general cognitive assessment tool [40]. Test were performed in Spanish and scores were normalised using Buenos Aires and Latin American norms, and classified as normal (N), mildly impaired (MI), or impaired (I) [41–43].

### MRI acquisition

All subjects underwent a standardised brain MRI protocol on a 3T scanner (Siemens Magneton Prisma, Erlangen, Germany) using a 64-channel head coil. The protocol included T1-MPRAGE, T2-FLAIR, resting state fMRI, diffusion tensor imaging (DTI), and arterial spin labelling (ASL). For this study, only the T1-MPRAGE image was used, which was acquired with the following parameters: TR = 2000ms, TE = 2.98ms, TI = 800ms, with a resolution of 1 × 1 × 1 mm. All scans were visually inspected for artefacts, and a radiologist reviewed the images for incidental findings. One participant was excluded due to an incidental finding, while two images were excluded from the analysis due to the presence of artefacts.

### MRI processing

Volumetric measures were obtained using two automated procedures for segmenting structural T1-weighted images: SIENAX of FSL (v6.0.7.7, http://www.fmrib.ox.ac.uk/fsl/) and FreeSurfer (v7.4 https://surfer.nmr.mgh.harvard.edu), both well documented and freely available.

Brain tissue volumes, normalized for intracranial volume, were estimated using SIENAX [44], part of FSL [45]. Measurements of total brain tissue volume, grey matter, white matter, peripheral grey matter, and ventricular CSF volumes were obtained.

Cortical reconstruction and volumetric segmentation were performed with the FreeSurfer image analysis suite. The technical details of these procedures are described in previous publications [46–48]. Briefly, this processing includes motion correction, removal of nonbrain tissue, automated Talairach transformation, and intensity normalisation. This software allows the labelling of regions on the cortical surface and subcortical brain structures. We extracted measurements of the volume of subcortical structures, as well as the volume and thickness of cortical regions using the Desikan-Killiany atlas.

All processed images were visually inspected, specially to confirm accurate brain extraction.

### Statistical analysis

For the standardised scales and cognitive assessment, Kruskal-Wallis and Chi-squared tests were performed to compare continuous and categorical variables between the two groups, respectively. Analysis of covariance (ANCOVA) was performed to compare cortical and subcortical grey matter volumes between groups, adjusting for age and total intracranial volume (TIV), as both factors are known to affect brain volume measurements. When these covariates were not significant, a two-sample t-test was used to directly compare the two groups. For cortical thickness measurements, a two-sample t-test was applied since TIV is not considered a relevant confounding factor. Statistical significance was set at *p* < 0.05.

Subcortical volume, cortical volume and cortical thickness analysis were corrected for multiple comparisons using the Westfall and Young step-down procedure [49] with 5000 permutations tests after regressing out the effects of age and ITV (when applicable). In this method, each permutation involves randomly shuffling group labels for each set of regional measurements and recalculating the t-statistic for each comparison (region), simulating the null hypothesis where no differences exist between groups. The t-statistics for both the observed data and each permutation are then ranked in descending order. The corrected p-values are calculated as the proportion of permutations where the threshold t-statistic exceeds the observed test statistic. The step-down procedure starts with the highest t-statistic from each permutation and progressively adjusts the threshold to the next highest values, as moving down to less significant findings. By adjusting p-values sequentially, this method balances control of the family-wise error rate while preserving statistical power to detect true effects.

Finally, Pearson correlation coefficients were calculated to explore the relationship between morphologic values, cognitive tests scores, and standardised questionnaire data continuous variables for the full sample and the LC group.

## Results

### Clinical characteristics

We recruited 109 subjects with long COVID symptoms (mean ± age 48.4 ± 8.0 years, 72.5% female) and 28 healthy controls (mean ± std age 45.2 ± 9.9 years, 70.4% female). The LC group reported persistent memory problems (83.5%), fatigue (78.9%), brain fog (79.8%) problems with attention (78.9%), muscle weakness (67.0%), muscle ache (50.5%), sleeping problems (49.5%), dyspnoea (47.7%), communication problems (44.0%) and headaches (40.4%). A considerable number of patients reported past symptoms of anosmia (40.4%) and loss of taste (38.4%), which were temporary.

All participants were clinically assessed to exclude other neurological problems. The LC group had the following medical histories: high blood pressure (30.4%), high cholesterol (25.7%), asthma (7.3%), diabetes (5.5%), angina pectoris (0.9%) and embolism or thrombosis (1.8%). The control group reported high blood pressure (25.9%), high cholesterol (29.6%), and diabetes (3.7%).

A detailed description of symptoms and other clinical data is presented in Table 1.

### Standardized scales

The LC group had higher levels of fatigue than the control group measured with the FAS scale (*p* < 0.01), with 59.6% classified as having fatigue, and 23.9% as having extreme fatigue. In terms of physical activity, 53.2% of the LC participants had low levels of physical activity compared to only 17.9% of the control group (*p* < 0.01). There were no significant differences in the PSQI test, as both groups had poor sleep quality.

In terms of health-related quality of life measured with the EQ-5D-5 L scale, only 1.8% (2 participants) of the LC group reported an ideal health status (no problems across all five dimensions) compared to 44.4% (12 participants) of the control group (*p* < 0.01). Health status with only slight (level 2) or no problem (level 1) in the five dimensions were reported by 40.3% of the LC group, compared to 78.8% of the controls. There was a significant difference in the self-reported health score (EQ VAS) between the groups, with the LC group scoring only 65.4 ± 17.4 compared to 80.9 ± 15.0 for the controls. The dimensions most affected in the LC group were pain/discomfort and anxiety/depression, which were reported more frequently than in the control group.

The full results for all standardised scales are shown in Table 2.

**Table 2:** Results of the standardized scales of fatigue (FAS), physical activity (IPAQ), sleep quality (PSQI) and health-related quality of life (EQ-5D-5 L)

		Control (28)	Long COVID (109)	*P* value
FAS, n(%)*	Extreme fatigue	0(0.0)%	26(23.9)%	**< 0.01**
	Fatigue	8(28.6)%	65(59.6)%	
	No fatigue	20 (71.4%)	17(15.6)%	
IPAQ, n(%)*	High	12(42.9)%	22(20.2)%	**< 0.01**
	Moderate	10 (35.7%)	28(25.7)%	
	Low	5(17.9)%	58 (53.2%)	
PSQI, n(%)*	Good	9 (32.1%)	18(16.5)%	0.09
	Poor	18 (64.3%)	91(83.5)%	
EQ-5D–5 L, n(%)* (No problems / slight problems / moderate problems / severe problems / extreme problems)	Mobility	24/3/1/0/0 (85.7% / 10.7% / 3.6% / 0% / 0%)	72/22/1/3/1 (66.1% / 20.2% / 9.2% / 2.8% / 0.9%)	0.38
	Self-Care	28/0/0/0/0 (100% / 0% / 0% / 0% / 0%)	98/7/3/0/0 (89.9% / 6.4% / 2.8% / 0% / 0%)	0.25
	Usual Activities	22/5/0/1/0 (78.6% /17.9% / 0% /3.6% / 0%)	56/32/12/7/1 (51.4% / 29.4% / 11.0% / 6.4% / 0.9%)	0.11
	Pain / Discomfort	18/6/4/0/0 (64.3% / 21.4% / 14.3% / 0% / 0%)	27/42/26/11/2 (24.8% / 38.5% / 23.9% / 10.1% / 1.8%)	**< 0.01**
	Anxiety / Depression	16/7/3/1/0 (57.1% / 25.0% /10.7% / 3.6% / 0%)	14/47/28/16/3 (12.8% / 43.1% / 25.7% / 14.7% / 2.8%)	**< 0.01**
	EQ VAS, mean ± SD**†**	80.9 ± 15.0	65.4 ± 17.4	**< 0.01**

* Chi-square test. † Kruskal-Wallis test

### Cognitive assessment

A reduced number of participants in the LC group exhibited impaired cognitive performance compared to normalised scales of the local population. The test most affected was the DGS-F, with 21.1% of the LC group showing impaired performance. Other tests with 10–15% of the LC group having impaired performance included the TMT-A, DGS-B, STROOP W, STROOP C and MoCA.

However, similar impairment patterns were observed in the control group and no significant differences were found in terms of performance and net scores between the two groups. Table 3 shows the net and normalised scores for each test, along with the percentage of participants classified as normal, mildly impaired, and impaired.

**Table 3:** Results of the cognitive assessment tests for the control and LC groups. Median(IQR) values are presented for net and normalised scores. The number and percentage of participants are shown for performance categories (normal, mildly impaired, and impaired). P-values correspond to the Kruskal-Wallis and Chi-squared tests, with no corrections applied for multiple comparisons

		Control (28)	Long COVID (109)	*P*-value
TMT - A	Score, median(IQR)	38.0(31.5–42.5)	41.0(33.0–51.0)	0.12
	Normalised score, median(IQR)	35.0(22.5–55.0)	25.0(10.0–50.0)	0.05
	Performance (A / MI / I)*	26(96.3%) / 1(3.7%) / 0(0.0%)	93(85.3%) / 4(3.7%) / 12(11.0%)	0.19
TMT - B	Score, median(IQR)	85.0(63.5–107.0)	85.0(68.0-112.0)	0.26
	Normalised score, median(IQR)	30.0(20.0-47.5)	30.0(15.0–55.0)	0.63
	Performance (A / MI / I)*	27(100.0%) / 0(0.0%) / 0(0.0%)	99(90.8%) / 5(4.6%) / 5(4.6%)	0.26
DGS – F (Digit Span Forward)	Score, median(IQR)	6.0(6.0–7.0)	7.0(6.0–8.0)	0.23
	Normalised score, median(IQR)	18.0(16.0–30.0)	22.0(17.0–51.0)	0.16
	Performance (A / MI / I)*	21(77.8%) / 0(0.0%) / 6(22.2%)	86(78.9%) / 0(0.0%) / 23(21.1%)	1.00
DGS - B (Digit Span Backward)	Score, median(IQR)	5.0(4.0–6.0)	5.0(4.0–7.0)	0.66
	Normalised score, median(IQR)	31.0(9.0–53.0)	30.0(12.0–54.0)	0.81
	Performance (A / MI / I)*	21(77.8%) / 4(14.8%) / 2(7.4%)	91(83.5%) / 4(3.7%) / 14(12.8%)	0.07
Stroop W	Score, median(IQR)	96.0(81.2–101.0)	92.0(80.0-105.0)	0.62
	Normalised score, median(IQR)	42.5(21.2–68.8)	40.0(10.0–75.0)	0.69
	Performance (A / MI / I)*	24(88.9%) / 0(0.0%) / 2(7.4%)	89(81.7%) / 5(4.6%) / 15(13.8%)	0.35
Stroop C	Score, median(IQR)	65.0(59.2–72.5)	63.0(54.0–70.0)	0.30
	Normalised score, median(IQR)	42.5(31.2–58.8)	35.0(15.0–65.0)	0.42
	Performance (A / MI / I)*	24(88.9%) / 2(7.4%) / 0(0.0%)	87(79.8%) / 7(6.4%) / 15(13.8%)	0.13
Stroop CW	Score, median(IQR)	41.0(33.5–45.8)	39.0(34.0–44.0)	0.61
	Normalised score, median(IQR)	47.5(20.0–70.0)	50.0(23.8–70.0)	0.85
	Performance (A / MI / I)*	25(92.6%) / 0(0.0%) / 1(3.7%)	103(94.5%) / 1(0.9%) / 5(4.6%)	0.87
Stroop Interf	Score, median(IQR)	1.5(-2.5-5.8)	1.0(-3.0-6.0)	0.76
	Normalised score, median(IQR)	60.0(36.2–75.0)	55.0(35.0–80.0)	0.57
	Performance (A / MI / I)*	25(92.6%) / 0(0.0%) / 1(3.7%)	104(95.4%) / 0(0.0%) / 5(4.6%)	1.00
MOCA	Score, median(IQR)	26.0(24.0–28.0)	26.0(24.0–27.0)	0.66
	Z score, median(IQR)	0.5(-0.4-1.1)	0.5(-0.1-0.8)	0.56
	Performance (A / MI / I)*	23(85.2%) / 3(11.1%) / 1(3.7%)	93(85.3%) / 5(4.6%) / 11(10.1%)	0.28

* A: Average, MI: Mildly Impaired, I: Impaired

**Fig. 2:**
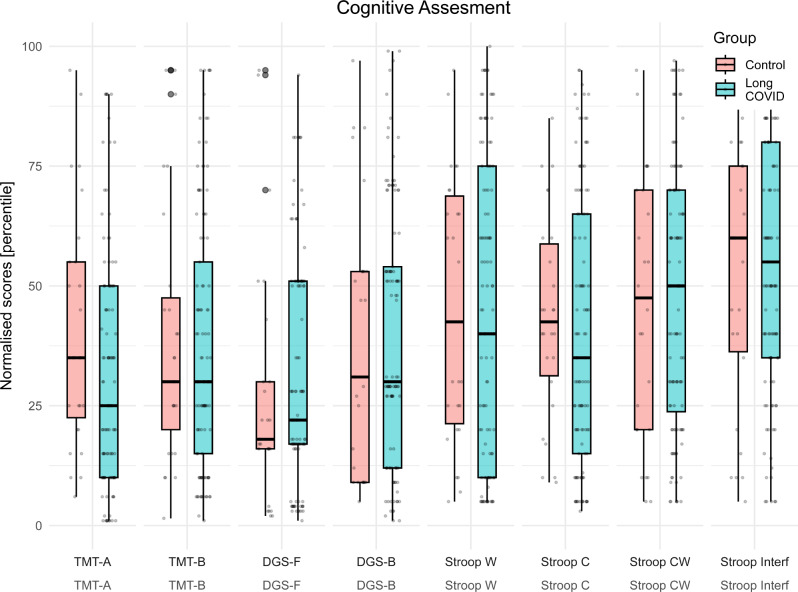
Box plots of normalised scores for each cognitive test for the control and LC groups. On each box, the central mark is the median, and the edges of the box are the 25th and 75th percentiles. The outliers are plotted individually with circles

When normalised scores by local scales were considered, the TMT-A showed lower performance for the LC group (*p* = 0.05). Figure 2 provides boxplots of the normalised scores for each test for each group.

### MRI processing

No significant differences in grey matter, white matter and total brain volume, normalised by intracranial volume and measured with Sienax, were found after controlling for age as a covariate.

The ANCOVA analysis of subcortical structures revealed a significant effect of long COVID on the volumes of the right cerebellum (*p* = 0.03) and left cerebellum (*p* = 0.03) after controlling for total intracranial volume and age. Significance was retained for the right cerebellum after correction for multiple comparisons using permutation tests. In the analysis of cortical regions, reduced grey matter volumes were observed in the left lingual gyrus and left inferior parietal lobule in the LC group; however, in this case, the significance did not survive correction for multiple comparisons. Table 4 presents the mean ± SD values for total brain volumes and regions with significant volumetric differences between the LC and control groups.

**Table 4:** Mean ± Std of brain volumes and the regions showing significant differences in volume and thickness between the LC and control groups. The F and p values from ANCOVA tests, and p-values from t-tests are presented. Additionally, p-values adjusted for multiple comparisons using permutations tests (p_mc_perm) are included

	Mean ± SD	Control (28)	Long COVID (109)	F	*p*-value	p_mc_perm
Volume [cm^3^]*	Grey Matter	777.1 ± 40.3	757.27 ± 39.47	2.95	0.09	-
	White Matter	760.7 ± 35.5	744.22 ± 39.41	2.50	0.12	-
	Total Brain	1537.8 ± 67.0	1501.49 ± 70.03	3.53	0.06	-
	Right Cerebellum Cortex	55.40 ± 4.75	52.60 ± 5.49	4.63	0.03	0.04
	Left Cerebellum Cortex	54.43 ± 4.33	51.64 ± 5.24	5.14	0.03	0.13
	Left Lingual Gyrus	6.74 ± 0.88	6.31 ± 0.99		0.04	0.17
	Left Inferior Parietal	12.30 ± 1.78	11.45 ± 1.75		0.03	0.19
	Right Inferior Temporal	11.00 ± 1.69	10.30 ± 1.52		0.04	0.19
Cortical Thickness [mm]†	Left Lingual Gyrus	2.03 ± 0.12	1.95 ± 0.10	-	< 0.01	0.03
	Left Postcentral Gyrus	2.12 ± 0.11	2.07 ± 0.09	-	0.02	0.03
	Left Superior Parietal Gyrus	2.27 ± 0.15	2.20 ± 0.09	-	0.05	0.05
	Left Precuneus	2.45 ± 0.12	2.38 ± 0.10	-	0.01	0.11
	Left Supramarginal Gyrus	2.54 ± 0.13	2.48 ± 0.10	-	0.03	0.03
	Left Banks of the Superior Temporal Sulcus	2.53 ± 0.15	2.44 ± 0.12	-	< 0.01	0.04
	Right Postcentral Gyrus	2.14 ± 0.14	2.07 ± 0.14	-	0.03	0.08
	Right Superior Temporal Gyrus	2.81 ± 0.10	2.76 ± 0.12	-	0.049	0.08
	Right Precuneus	2.45 ± 0.10	2.40 ± 0.10	-	0.02	0.11

* ANCOVA analysis. † T-tests

For the cortical thickness, the LC group exhibited reduced thickness in several regions, including the right postcentral gyrus (*p* = 0.03), superior temporal gyrus (*p* = 0.049), and precuneus (*p* = 0.01); as well as the left banks of the superior temporal sulcus (*p* < 0.01), lingual gyrus (*p* < 0.01), postcentral gyrus (*p* = 0.02), precuneus (*p* = 0.01), and supramarginal gyrus (*p* = 0.03). The significance survived correction for multiple comparisons in five of the mentioned regions. The p-values for the t-tests along with the p-values corrected for multiple comparisons with Westfall and Young permutation tests method (p_mc_perm) are presented in Table 4.

**Fig. 3:**
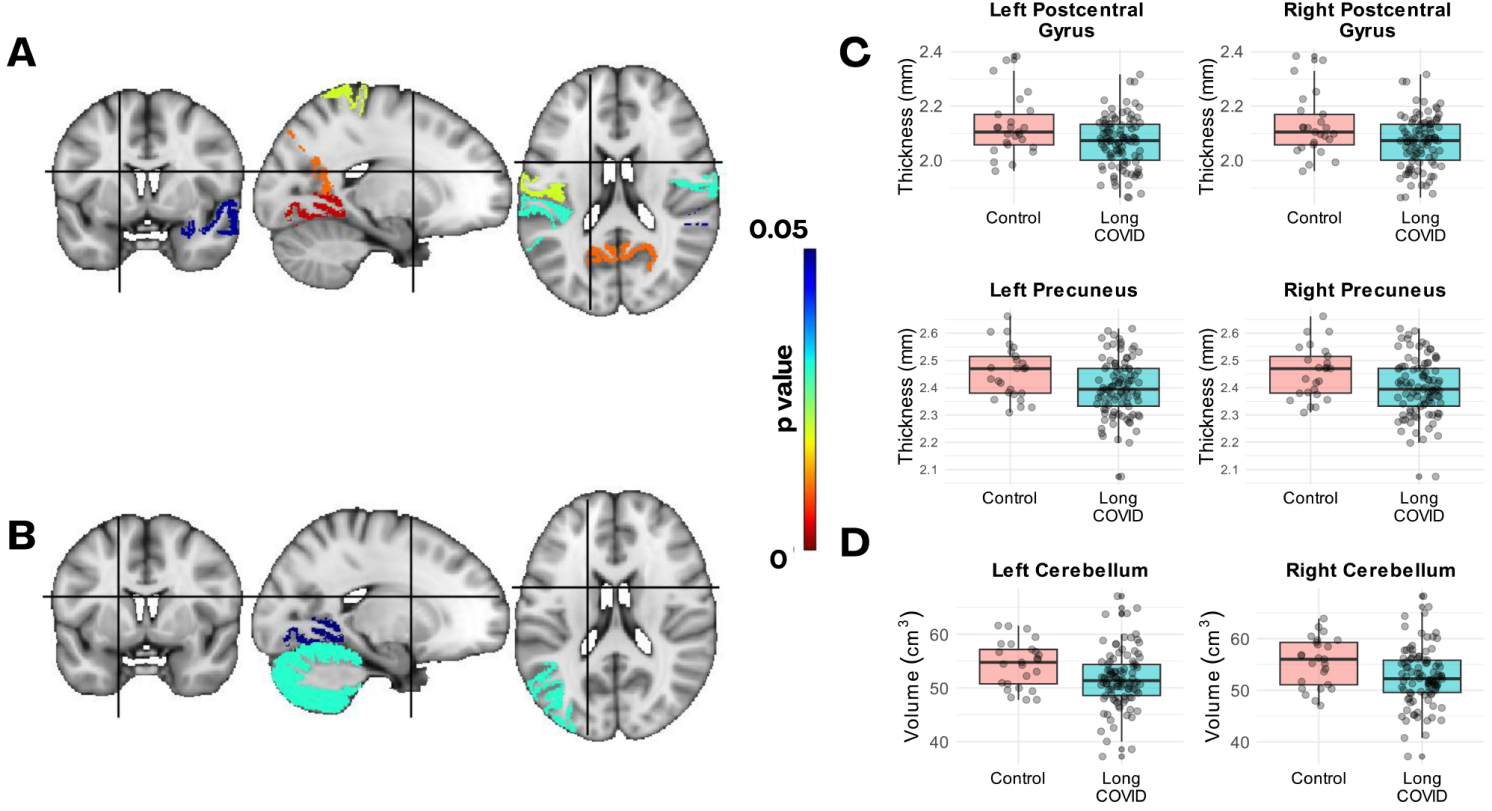
Regions of the Desikan-Killiany atlas with signs of atrophy in the LC group. Regions with differences between the LC and controls groups in their cortical thickness (**A**) and volume (**B**) are highlighted with a colorbar as a function of the raw p-value (uncorrected for multiple comparisons). **C**) Boxplots of the cortical thickness of the left and right postcentral gyrus and precuneus for the control and LC groups (shown in **A**). **D**) Boxplots of left and right cerebellum grey matter volume. On each box, the central mark is the median, and the edges of the box are the 25th and 75th percentiles

Figure 3 shows regions with significant differences in cortical thickness (A) and volume (B) between the LC and control groups, displayed in MNI152 space. Boxplots for the regions with significant differences in thickness (C) and volume (D) on both hemispheres are also shown.

No significant correlations were found between regions with signs of atrophy and the results of the cognitive tests scores and the standardised questionnaire. Correlation matrices with the Pearson correlation coefficients between the main variables are shown in Supplementary Fig. 1 for the full data sample and in Supplementary Fig. 2 only for the LC group.

## Discussion

This study provides valuable insights into the cognitive impact and potential brain structural changes in patients with long COVID approximately two years post-infection (908 days in average). This is one of the few studies focusing on the impact of long COVID in Latin America populations, a region where this condition has been greatly overlooked. We found that the individuals of the LC group reported a variety of persistent symptoms, particularly in cognitive domains, which align with research conducted on other populations. The standardised scales revealed higher levels of fatigue and reduced quality of life in the LC group compared to healthy controls. Interestingly, although the cognitive evaluations showed a reduction in performance using local scales, the differences were not statistically significant compared to the control group, except for the TMT-A test. While no clear cognitive differences were found between the LC and control groups, we did found differences in brain morphology between them.

### Cognitive impairment in long COVID

Despite participants reporting multiple persistent cognitive symptoms, our cognitive tests did not indicate significant cognitive decline. This discrepancy suggests that the cognitive tests used may not be sensitive enough to detect subtle impairments, or that the subjective cognitive complaints may be influenced by other daily life factors.

Our results contrast with several studies that have demonstrated cognitive deficits in areas such as attention, executive function, and memory shortly after COVID-19 infection. For example, Douaud et al. found cognitive impairment in patients measured by the completion time of TMT-A and TMT-B, on average 141 days post-infection [12]. Notably, TMT-A was the only test in our study with marginally significant differences, suggesting that specific tasks might be more susceptible to impairment in long COVID.

Recent studies suggest cognitive decline may improve over time. For example, a longitudinal study demonstrated that COVID-19 survivors with significant cognitive decline 6 months post-infection showed improvement one year later, indicating possible spontaneous recovery [50]. Huang et al. also noted a pattern of cognitive decline and recovery in COVID-19 patients [51]. Furthermore, a more recent study found that cognitive deficits may have attenuated as the pandemic progressed, as they found smaller cognitive deficits in participants infected during recent variant periods [52]. In our study, despite recruiting participants infected with COVID-19 at any time since the start of the pandemic, most patients with persistent symptoms were infected during the pandemic’s first and second years.

Similar findings have been reported by Walitt et al. for post-infectious myalgic encephalomyelitis / chronic fatigue syndrome [53], a condition that shares many similarities with long COVID [54], where they observed differences in perception, but no significant differences in neurocognitive standardized tests.

### Structural brain changes

Structural MRI analysis revealed decreased volumes in the left and right cerebellum, left lingual gyrus, and left inferior parietal regions. However, significance survived for multiple comparisons only for the right cerebellum. The reduction in cerebellar volume aligns with longitudinal studies that observed cerebellar volume reduction in recovered COVID-19 patients at a 2-year follow-up [55]. Hypometabolism in the cerebellum has also been reported using ^18^F-FDG PET, indicating metabolic changes in this region [56]. The cerebellum’s role in executive functions and working memory may explain some of the cognitive symptoms in long COVID [57].

Additionally, cortical thickness reduction was noted in several regions, including the right and left postcentral, superior temporal gyrus and precuneus, which are also affected in Alzheimer’s disease [58, 59]. Although only a subset of these regions remained significant after correction for multiple comparisons, the overall pattern of reduction suggests a clear trend, underscoring the importance of studying the long term effects of long COVID to determine whether it may increase the risk of neurodegenerative diseases in the future.

When assessing structural differences in relation to cognitive performance, we did not find any significant correlations. However, it is possible that these structural alterations could precede or occur independently of observable cognitive deficits. Cognitive processes are complex and do not directly align with structural brain changes [60]. In addition, the structural changes we observed may not directly relate to the cognitive functions assessed, or the tests used may lack sensitivity to detect subtle correlations, especially given the varying levels of cognitive reserve among participants.

Moreover, the structural changes we detected could be related to other symptoms or lifestyle changes associated with long COVID. For example, patients with chronic fatigue syndrome have exhibited decreased blood oxygen level-dependent (BOLD) signals in the superior parietal lobe and right temporal gyrus, regions where we also observed structural differences between groups. This suggests that these structural alterations may reflect broader systemic effects beyond cognitive function alone.

### Quality of life and physical activity

We found a significant reduction in health-related quality of life and physical activity levels in the LC group. These results show a persistent impact of long COVID symptoms on quality of life even two years post-infection. While our recruitment specifically targeted individuals experiencing cognitive symptoms, it is important to note that all participants reported multiple cognitive issues simultaneously, including symptoms such as brain fog, communication difficulties, memory problems, and attention deficits.

The EQ-5D-5 L scale indicated more problems in pain/discomfort and anxiety/depression dimensions, underscoring the need for interventions addressing both physical and mental health to improve overall well-being.

### Long-term impact of long COVID

Our study indicates that two years post-infection, people with long COVID symptoms continue to experience a wide range of symptoms, significantly affected quality of life, mild signs of brain atrophy, but no clear cognitive decline.

As a cross-sectional study, we could not assess changes over time or infer if cognitive function improved as suggested by other studies. However, a small group showed cognitive compromise, supporting the need for longitudinal studies to follow long COVID patients and assess their risk for long-term neurodegenerative diseases.

### Limitations

Our study has some limitations. First, the cross-sectional design limits our ability to infer causality of the differences between groups (i.e., the differences between groups are a direct result of long COVID). Second, although the sample size is large for an imaging study, it may still have been insufficient, particularly for the control group, to detect small but clinically significant differences. However, this study represents one of the largest neuroimaging cohorts of long COVID patients, and the use of permutation testing to assess structural brain differences offers a robust safeguard against potential biases introduced by the sample size imbalance.

Lastly, participants were recruited based on self-reported persistent symptoms after a COVID-19 infection, a common challenge in long COVID studies due to the lack of specific diagnostic tools or biomarkers. However, we conducted extensive clinical evaluations to exclude participants with other clinical conditions.

## Conclusions

This study highlights the persistent symptoms and potential brain structural changes in long COVID patients two years post-infection in a cohort from Argentina. Although cognitive tests did not show significant impairment, structural MRI revealed specific regions of brain atrophy. The substantial reduction in quality of life and physical activity levels in the LC group underscores the need for comprehensive interventions addressing both physical and mental health aspects. Given these findings, longitudinal studies are essential to understand the progression and long-term impact of long COVID on cognition and brain health, and to develop effective therapeutic strategies to support affected individuals.

## Electronic supplementary material

Below is the link to the electronic supplementary material.

Supplementary Material 1

## Data Availability

Part of the data is available from the corresponding author on reasonable request.
