# Supplementary material for: Cognitive impact and brain structural changes in long COVID patients: a cross-sectional MRI study two years post infection in a cohort from Argentina
Source: BMC Neurol. 2024 Nov 18;24:450. doi: 10.1186/s12883-024-03959-8 (PMC11572126; doi:10.1186/s12883-024-03959-8)
Supplement: Supplementary file 1 — Supplementary Material 1 [file 12883_2024_3959_MOESM1_ESM.docx]

**Supplementary Material**


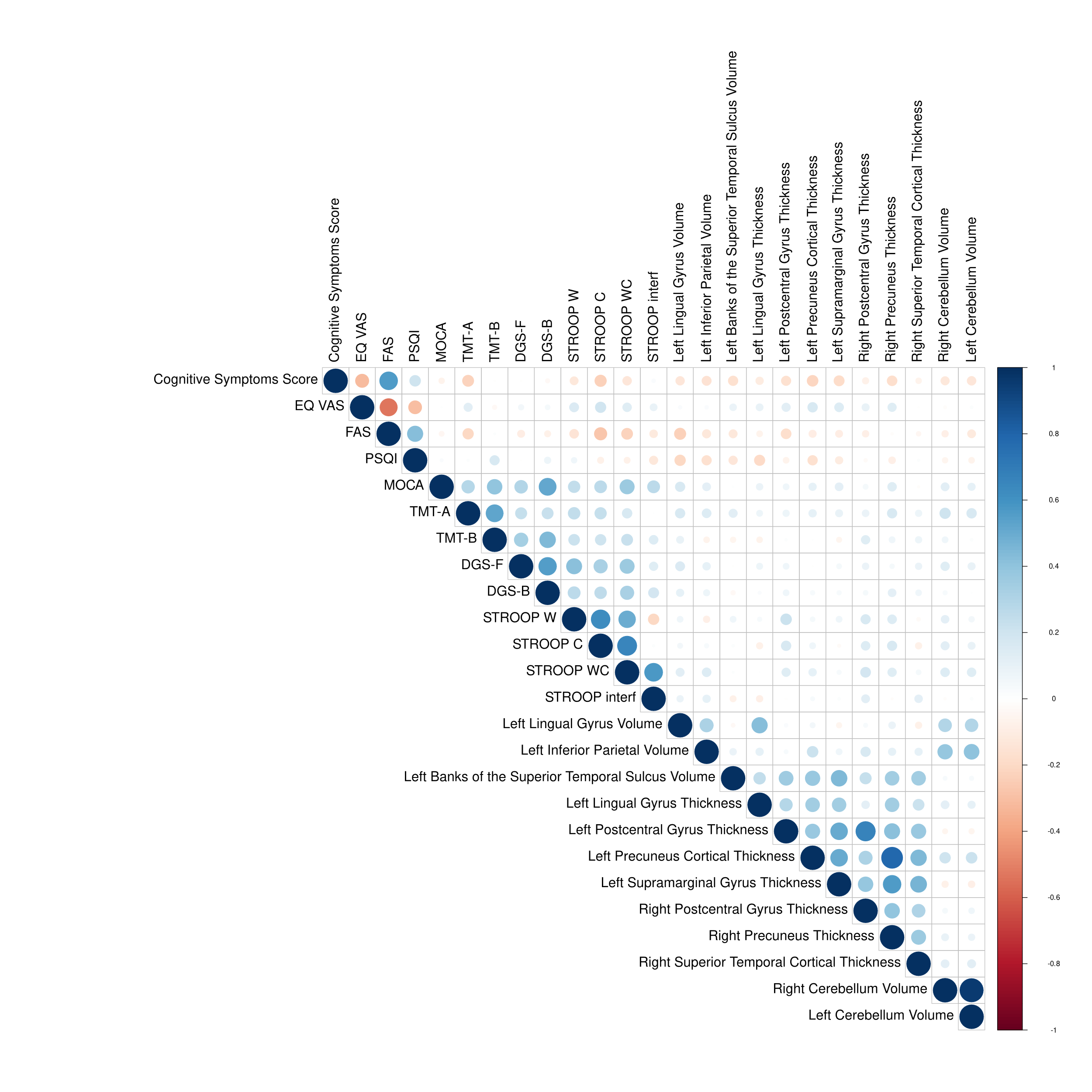


Supplementary Figure 1. Correlation matrix with the Pearson correlation coefficient between the scores of the cognitive tests, the main results of the standardised questionnaires and the brain regions with signs of atrophy in the LC group, computed for the full sample (LC and control groups) data.


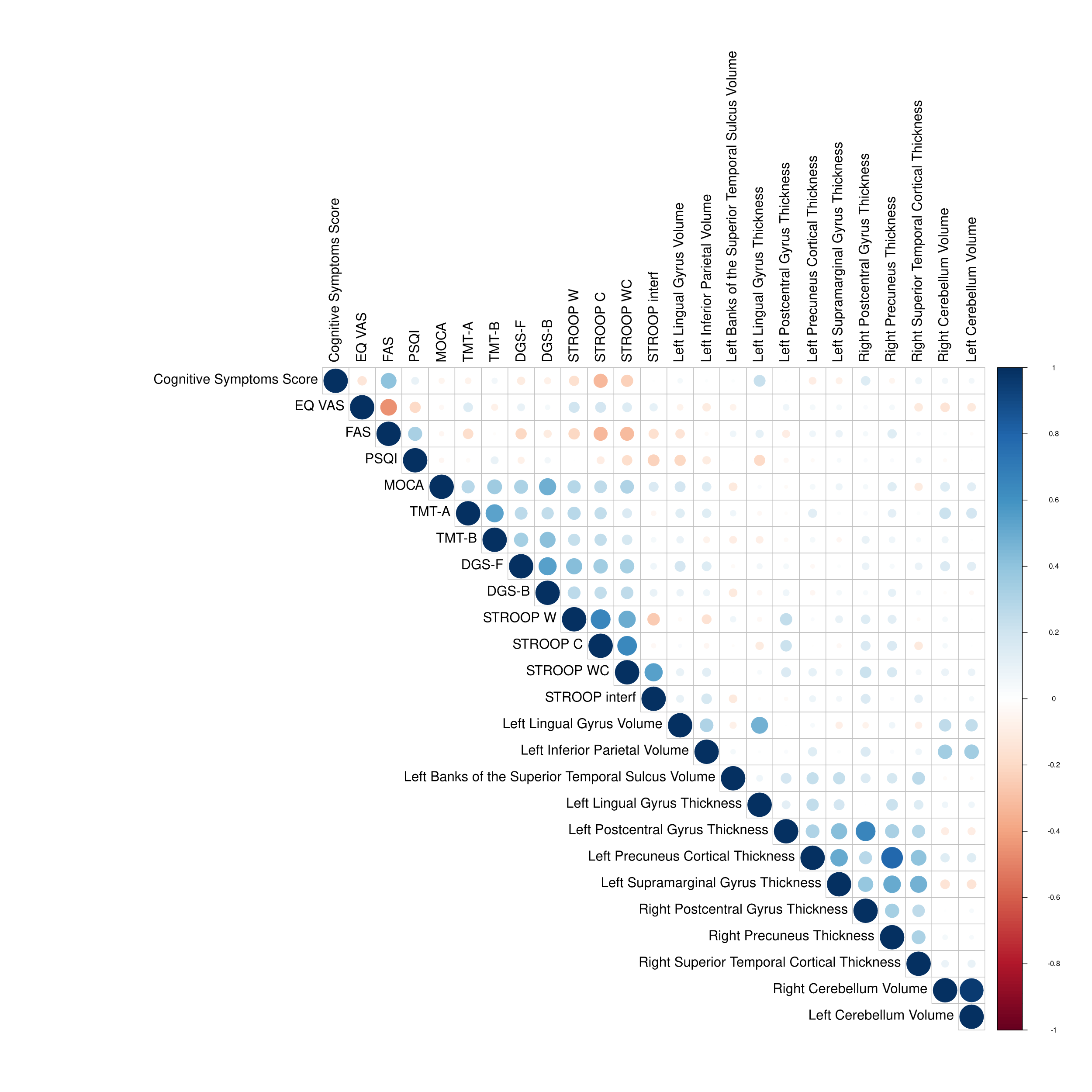


Supplementary Figure 2. Correlation matrix with the Pearson correlation coefficient between the scores of the cognitive tests, the main results of the standardised questionnaires and the brain regions with signs of atrophy in the LC group, computed for the LC group data.
